# Time-Trends of Drug-Drug Interactions among Elderly Outpatients in the Piedmont Region (Italy): A Population-Based Study

**DOI:** 10.3390/ijerph19127353

**Published:** 2022-06-15

**Authors:** Elisabetta Galai, Lorenza Scotti, Marco Gilardetti, Andrealuna Ucciero, Daniela Ferrante, Elisabetta Poluzzi, Armando A. Genazzani, Francesco Barone-Adesi

**Affiliations:** 1 Department of Pharmaceutical Sciences, Università del Piemonte Orientale, 28100 Novara, Italy; elisabetta.galai@maggioreosp.novara.it (E.G.); armando.genazzani@uniupo.it (A.A.G.); 2 Azienda Ospedaliero-Universitaria “Maggiore della Carità”, 28100 Novara, Italy; 3 Department of Translational Medicine, Università del Piemonte Orientale, 28100 Novara, Italy; andrealuna.ucciero@uniupo.it (A.U.); daniela.ferrante@med.uniupo.it (D.F.); francesco.baroneadesi@uniupo.it (F.B.-A.); 4 SC Epidemiologia dei Tumori CRPT U, Azienda Ospedaliero-Universitaria (A.O.U.) Città della Salute e della Scienza di Torino, Centro di Riferimento per l’Epidemiologia e la Prevenzione Oncologica (CPO) Piemonte, 10100 Torino, Italy; marco.gilardetti@cpo.it; 5 Department of Medical and Surgical Sciences, Alma Mater Studiorum, University of Bologna, 40100 Bologna, Italy; elisabetta.poluzzi@unibo.it

**Keywords:** elderly, drug-drug interactions, time trend

## Abstract

Adverse drug reactions (ADRs) are a major health problem in the primary care setting, particularly among the elderly population. While the high frequency of ADRs in the elderly has several causes, a major and common determinant is polypharmacy, which can in turn increase the risk of drug-drug interactions (DDIs). In this paper, we analyzed the drugs prescriptions dispensed to elderly outpatients, to assess changes in the prevalence of selected DDIs in the period 2013–2019. Overall, about 15% of the patients aged >65 years were poly-treated. Among them, a decreasing trend in prevalence was observed for the majority of DDIs during the study period. This trend was particularly noticeable for DDIs involving fluoroquinolones and vitamin K antagonists, where a sharp reduction of over 40% was observed. On the opposite, a small increase in prevalence was observed for the association of antidiabetics and beta-blocking agents and for that of clopidogrel and PPIs. While the occurrence of most of the considered DDIs among poly-treated elderly decreased over time, the prevalence of some of them is still worrying. The complexity of the national drug formularies, as well as the increased number of prescribing actors that are involved, further urges the update of DDI lists to be used to monitor drug appropriateness and reduce avoidable ADRs.

## 1. Introduction

Adverse drug reactions (ADRs) are a major health problem in the primary care setting as they correlate with morbidity and mortality [1,2,3,4], and this is even more relevant in the elderly (≥65 years) population [5,6] where over a quarter of the patients have been reported to suffer from ADRs [7]. Similarly, an analysis of the World Health Organisation’s pharmacovigilance database reported that the highest rate (33.3%) of fatal ADRs is found in those over 75 years of age [4].

While the high risk of ADRs in the elderly has several causes, including frailty, comorbidities, and different degrees of physiological or pathological hepatic and renal impairment [8,9], a major and common determinant is due to polypharmacy. For example, the most recent Italian National drug report shows that the average drug consumption for the entire Italian population is approximately 1 defined daily dose (DDD) per inhabitant, but it rises to 2 in the 65–69 years-old patients and to 2.5 in the 70–74 years-old patients, exceeding a value of 3 in those over 75 years [10]. Although in most cases polypharmacy is necessary to treat multiple coexisting conditions, it is acknowledged that this also increases the probability of drug-drug interactions (DDIs), in which co-administered drugs may negatively interact at either the pharmacokinetic or pharmacodynamic level [11,12,13]. A high prevalence of DDIs in poly-treated patients have been reported by several authors. The MULTIPAP study, carried out in Spanish primary health care centres on multi-treated (≥5 drugs) elderly patients (65–74 years) with multimorbidity (≥3 chronic diseases), showed that half of the patients had at least one DDI in their prescriptions [14]. Similarly, a cross-sectional study carried out in Brazil reported that the prevalence of DDI among elderly patients (≥60 years) who were poly-medicated was around 35% [15]. The impact of DDIs on the population rates of ADRs can be considerable. Indeed, it has been estimated that between 6% and 30% of all ADRs in the population are due to DDIs, and as such could be largely avoidable [16,17]. Constant monitoring at patient and/or population level of inappropriate prescriptions that may lead to DDIs is therefore an important activity to prevent ADRs.

Although the importance of DDIs in the onset of ADRs cannot be overemphasized, to date only few studies have evaluated time trends of DDI prevalence among elderly in the primary care setting, both in Italy [18,19] or elsewhere [20,21]. Overall, all these studies report that the prevalence of DDI remained fairly stable or increased over time, despite the implementation of several information and education campaigns directly targeted to health professionals.

In the present paper, we analyzed the prescriptions of reimbursed drugs that were dispensed to elderly outpatients in the Piedmont region (Italy), to assess changes in the prevalence of polypharmacy and of selected DDIs between 2013 and 2019.

## 2. Materials and Methods

### 2.1. Data Sources

The current study is based on the data retrieved from the healthcare utilization database of the Piedmont region (over 4 million inhabitants, corresponding to 7.5% of the Italian population). The drug reimbursed by the Italian National Health System dispensed by hospital and local pharmacies extracted from this database were linked to the inhabitants’ registry containing sex and age through an encrypted identification code.

### 2.2. Setting

The target population consisted of all subjects aged 65 years or older residing in Piedmont between 2013 and 2019 treated with polytherapy. Polytherapy was defined as the concomitant use of at least five therapeutic classes (defined at the fourth level of the ATC code) each with a coverage of at least 90 days within the first 6 months of the year [18,22]. For each year considered, subjects alive on the 1st of January were included in the cross-sectional observation.

### 2.3. Evaluation of Drug-Drug Interactions

Raschi et al. identified 53 DDIs at the fourth or fifth ATC level and evaluated their prevalence in the Emilia Romagna Region between 2011 and 2013 [18]. From this list, we focused on DDIs with a reported prevalence above 5%, namely: (i) antidiabetics and beta- blocking agents; (ii) antidiabetics and fluoroquinolones; (iii) angiotensin-converting-enzyme inhibitors (ACEIs) or angiotensin II receptor blockers (ARBs) and non-steroidal anti-inflammatory drugs (NSAIDs); (iv) ACEIs or ARBs and potassium-sparing agents; (v) ACEIs or ARBs in combination with diuretics and NSAIDs; (vi) diuretics and NSAIDs; (vii) selective serotonin reuptake inhibitors (SSRIs) and NSAIDs or acetyl salicylic acid (ASA); (viii) vitamin K antagonist and proton pump inhibitors (PPIs); (ix) vitamin K antagonists and statins; (x) clopidogrel and PPIs; and (xi) corticosteroids and NSAIDs or ASA. For each year, all the prescriptions of the drugs of interest were identified and the coverage of each prescription was calculated according to their defined daily dose. The full list of the ATC codes is reported in the Supplementary Materials (Table S1). A subject was defined as experiencing a specific DDI if the coverage of the drugs involved in the DDI overlapped for at least one day.

### 2.4. Statistical Analysis

Descriptive statistics were used to summarize the main characteristics of the subjects. Categorical variables were reported as absolute frequencies and percentages while continuous variables as mean and standard deviation (SD). For each year, the prevalence of each DDI, expressed as percentage, was calculated as the ratio between the number of subjects experiencing the DDI and the number of poly-treated subjects. The prevalence was calculated on the entire sample and stratified by sex and age classes (65–69 years, 70–74 years, 75–79 years, 80–84 years, and ≥85 years). The relative change in prevalence of the DDIs between 2013 and 2019, calculated as the difference in prevalence between 2013 and 2019 divided by the prevalence in 2013, and the corresponding 95% confidence intervals (95% CI) were also calculated for the overall sample. All analyses were performed using SAS 9.4 (SAS Institute, Inc., Cary, NC, USA).

## 3. Results

**Figure 1:**
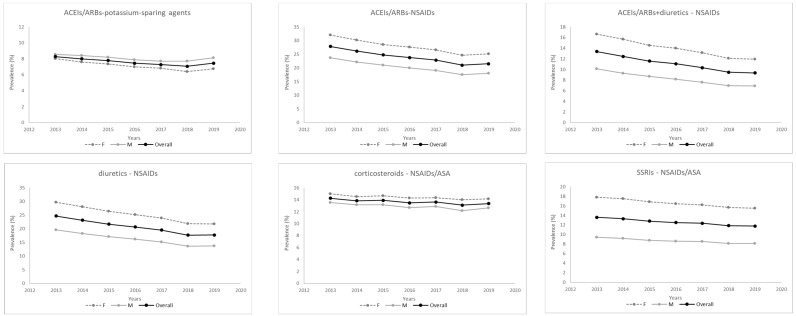
Trend in prevalence of DDIs involving antihypertensives and/or NSAIDs overall and by sex. 2013–2019, Piedmont region (Italy).

Table 1 shows the main characteristics of the included subjects by year. While the overall population size saw a slight decrease over time, the proportion of older subjects (≥65 years) and those poly-treated slightly increased along the study period. Polypharmacy, defined as subjects prescribed to at least five different therapeutic classes at a given time, increased from 13.49% of the ≥65 population in 2013 to 14.99% in 2019. No relevant changes over time were observed regarding sex distribution and age, reported both as mean and age classes. The prevalence of the analyzed DDIs is represented in Figure 1 and Figure S1 (Supplementary Materials) and the relative change in prevalence between 2013 and 2019 is reported in Table 2.

Overall, a decreasing trend in prevalence over time was observed for all DDIs, except for antidiabetics and beta-blocking agents and for clopidogrel and PPIs, for which an increase of 10.94% (95% CI 9.57%; 12.33%) and 14.36% (11.67%; 17.12%) was observed, respectively (Table 2). DDIs involving fluoroquinolones or vitamin K antagonists showed the highest decrease, with a reduction of about 50%. The decreasing trend was also particularly marked for diuretics and NSAIDs (relative change: −28.19%, 95% CI −29.14; −27.24) and ACEIs/ARBs and NSAIDs (percent change: −22.70%, 95% CI −23.62%; −21.77%), Regarding the stratification by sex, the time trends in men and women were similar but men had a higher prevalence of DDIs for clopidogrel and PPIs (8.38% vs. 5.80% in 2013) as well as vitamin K antagonists and statins (9.77% vs. 6.99% in 2013) while no substantial difference between genders were observed for ACEIs/ARBs and potassium-sparing agents (men 8.54% vs. women 8.02% in 2013), corticosteroids and NSAIDs/ASA (men 13.51% vs. women 14.97% in 2013), vitamin K antagonists and PPIs (men 11.20 % vs. women 10.30% in 2013), antidiabetics and both beta-blocking agents (men 22.26% vs. women 21.43% in 2013), and fluoroquinolones (men 11.33% vs. women 10.48% in 2013). The yearly prevalence stratified by age classes are reported in Table 3 (DDIs involving antihypertensives and/or NSAIDs) and Table S2 (Other DDIs).

In the first years of our analysis (2013–2016), older age was associated with a reduced prevalence of co-prescription of ACEIs/ARBs with diuretics and NSAIDs, but then this difference blunted due to a decreased co-prescription in the younger age groups. On the other hand, the trend remained when evaluating only the prescriptions of ACEIs/ARBs and NSAIDs. Conversely, in 2013 there was an increased prescription of SSRIs and NSAIDs/ASA that correlated with age that blunted in later years.

## 4. Discussion

In the present study, we used administrative data from a large Italian region to evaluate changes in the prevalence of polypharmacy and selected DDIs over a 7-year period.

We found that in 2019 about 15% of the elderly residents used at least five different therapeutic classes, which corresponds to an absolute increase of about 1.5% from 2013. These results are similar to those by Raschi et al., reporting 15.2% of Emilia-Romagna residents prescribed more than five drug classes in 2011 and 16.7% in 2013 [18]. The increase over time of polypharmacy that we observed was probably due to the expansion of the drug formulary and the consequent increase of the available therapeutic options, although changes in the age structure of the elderly population could also have played some role. Overall, our data further strengthen the notion that polypharmacy is common among the elderly.

Regarding the prevalence of specific DDIs, the five most frequent ones that we observed in the Piedmont region are the same that Raschi et al. reported for the Emilia-Romagna region in the first semester of 2013 [18]. However, we also found a substantial reduction of prevalence over time for most of the considered DDIs. In particular, the co-prescription of fluoroquinolones and antidiabetics, of vitamin K antagonists and PPIs, and of vitamin K antagonists and statins decreased by over 40% over the study period. We assume that the main reason for this is attributable to the changing landscape in therapeutic strategies that occurred during the study period, rather than to an increasing awareness of prescribers about this DDIs. Fluoroquinolones have been recently subject to safety scrutiny and in 2018 both FDA [23] and EMA [24] issued safety warnings, which translated into a subsequent restriction or suspension of several drugs belonging to this class. The effect of these warnings is evident in our data in 2019 and it is in line with the observed change in prescribing patterns in the US following regulatory changes due to safety re-assessment of this class of antibiotics [25]. Nevertheless, we note that the use of fluoroquinolones in Piedmont was still higher than expected even in 2019, considering the reported health risks and the availability of alternatives. This suggests that the inappropriate use of this class of antibiotics is still relatively common.

We also observed a decrease in DDIs involving vitamin K antagonists. In our opinion, this could be mainly due to the gradual transition from warfarin to new oral anticoagulants, such as dabigatran and rivaroxaban, which occurred in the last decade. Thus, also in this case changes in prescribers’ awareness towards DDIs probably played a limited role in the observed results.

A second cluster of potential DDIs saw a relative decrease of around 30%, namely those involving NSAIDs and hypertensive drugs working on the renin-angiotensin system, either in presence or absence of diuretics. Co-administration of these drugs is postulated to trigger pharmacodynamic interactions in which the hypotensive effect of ACEIs/ARBs is antagonized by NSAIDs and there is also an increased risk of renal failure in the presence of diuretics [18]. It should be noted that in the elderly population, owing to a physiological liver and kidney impairment, even the chronic use of NSAIDS alone is considered inappropriate [18,26,27,28] because of the increased risk of gastrointestinal toxicity, renal side effects, and cardiovascular events [9,29,30,31,32]. The reduction in the use of NSAIDs in the elderly population is also the most likely explanation for the observed reduction of in the co-prescription of SSRIs and NSAIDs/ASA.

During the study period, two DDIs showed a slightly increase, namely antidiabetics and beta-blocking agents and clopidogrel and PPIs. As for the DDI involving antidiabetics and beta-blocking agents, this may provide an example of two drugs deliberately and unavoidably prescribed together [33]. In the Italian population, beta-blocking agents represent one the most frequently prescribed drug classes, with a prevalence of use of around 25% in patients between 65 and 74 years and an even higher one among those older than 85 years (about 40%) [27]. Yet, beta-blocking agents may trigger unrecognized hypoglycemic crisis and impair glycemic control, thereby affecting the action of antidiabetic drugs. While this DDI is still considered important, newer beta-blocking agents seem to have a reduced effect on glycemic control. This suggests that educational campaigns to guide clinicians in choosing these newer beta-blockers and/or to inform them of the importance of monitoring glucose levels more frequently in patients using both medications could be more effective than recommending the avoidance of such combinations altogether [34]. Given that clopidogrel is a prodrug that is activated by CYP2C19 and that most PPIs are inhibitors of this enzyme, it has been suggested that their co-administration may result in an increased thrombotic risk [35]. However, the pharmacodynamic interaction between PPIs and clopidogrel depends on the potency of each PPI to inhibit CYP2C19 [36] and therefore not all the co-prescriptions would significantly increase patient risk. On the other hand, it has been observed that PPIs, which are frequently prescribed in poly-treated patients and in those with comorbidities, are often inappropriately prescribed and even involved in ADRs themselves, posing questions to their real necessity [37,38]. Indeed, reducing the inappropriate prescription of PPIs is still an important health policy issue [39,40,41].

Overall, our data suggest that most DDIs that had been a focus of pharmacoepidemiological studies and educational campaigns in the last decade now have a reduced prevalence and impact, either because clinicians pay more attention to these DDIs or because newer and safer drugs have emerged on the market, replacing those involved in DDIs.

This underlines the importance of periodically updating DDIs to be monitored to reduce the risk of ADRs. To the same aim, strategies aimed to avoid DDI such as minimizing the number of drugs prescribed by physicians, considering non-pharmacological treatment options, or adjusting drugs’ dosage, among others, should be implemented [42].

Outcome studies at the local level can support periodical updates of DDI lists as well educational campaigns to reduce them. For instance, Swart et al. recently used data from a different Northern Italian area and found an increase in hospitalization due to DDIs involving antidiabetics-fluoroquinolones, NSAIDs- vitamin K antagonists, and NSAIDs- SSRIs. The decrease of these co-prescriptions shown by our work thus could represent a favorable finding also in terms of clinical outcomes [43].

### Strength and Limitations

The present contribution has as its major strength the long observation time (7 years) and the use of the same list of DDIs proposed in previous studies [18], thus making comparisons with published research straightforward. A further strength is given by the large dataset examined, which included over a million elderly inhabitants. Some weaknesses of the study should be highlighted as well. First, the data used came from administrative databases and focused on DDIs that are known or suspected to increase the risk of ADRs. However, as we did not collect clinical data, we cannot estimate in which proportion of patients ADRs actually occurred. Regarding this, it should be also underlined that the risk and the severity of ADRs can be very variable depending on the DDIs considered and on the dosage of the drugs involved in the DDI. Second, to align the definitions to what was done previously, most drug classes were defined at the fourth ATC code, thereby ignoring the differences that indeed exist within some classes of considered drugs, such as PPIs and beta-blocking agents. This limitation can have overestimated the actual prevalence of relevant DDIs. On the other hand, as our database included only drugs reimbursed by the Italian National Health System, DDIs involving medications that can also be purchased out-of-pocket (e.g., NSAIDs) were probably underestimated. Last, we extracted DDIs but did not correlate this with the overall use of the single classes, which is a plausible explanation for some observed changes (e.g., reduced used of fluoroquinolones and warfarin). Finally, while the present approach allowed us to include a large number of known DDIs, it should be kept in mind that our knowledge on possible inappropriate uses of medications increases every day. New methods based on the evaluation of drugs (in particular psychotropics) according to their receptor affinity hold the promise of better defining inappropriate prescribing in the near future [44].

## 5. Conclusions

While the occurrence of most of the considered DDIs among poly-treated elderly decreased over time, the prevalence of some of them is still worrying, also considering the concurrent increase of polypharmacy in the population. The complexity of the national drug formularies, as well as the increased number of prescribing actors that are involved, further urges the update of DDI lists to be used to monitor drug appropriateness and reduce avoidable ADRs.

## Figures and Tables

**Table 1:** Main characteristics of study subjects by year. 2013–2019, Piedmont region (Italy).

	Year						
	2013	2014	2015	2016	2017	2018	2019
Subjects, N	4,924,918	4,874,715	4,825,135	4,771,202	4,719,997	4,666,102	4,612,679
Over 65, N (%)	1,146,111 (23.27)	1,160,271 (23.80)	1,173,887 (24.33)	1,180,494 (24.74)	1,190,234 (25.22)	1,196,981 (25.65)	1,205,837 (26.14)
Polypharmacy, N (%)	154,584 (13.49)	162,809 (14.03)	166,984 (14.22)	173,534 (14.70)	177,804 (14.94)	179,669 (15.01)	180,750 (14.99)
Males, N (%)	491,701 (42.90)	499,622 (43.06)	507,160 (43.2)	511,591 (43.34)	517,580 (43.49)	522,214 (43.63)	527,763 (43.77)
Age, mean (SD)	76.21 (7.97)	76.29 (8.04)	76.4 (8.11)	76.5 (8.16)	76.61 (8.22)	76.7 (8.28)	76.78 (8.34)
Age Class, N (%)							
65–69 years	279,275 (24.37)	286,799 (24.72)	297,187 (25.32)	291,655 (24.71)	285,931 (24.02)	279,039 (23.31)	277,388 (23.00)
70–74 years	259,228 (22.62)	250,269 (21.57)	238,264 (20.30)	244,551 (20.72)	253,199 (21.27)	263,515 (22.01)	270,870 (22.46)
75–79 years	234,410 (20.45)	241,058 (20.78)	245,690 (20.93)	245,772 (20.82)	242,620 (20.38)	235,066 (19.64)	226,956 (18.82)
80–84 years	184,095 (16.06)	185,598 (16.00)	186,361 (15.88)	186,833 (15.83)	190,430 (16.00)	197,028 (16.46)	203,225 (16.85)
≥85 years	189,103 (16.50)	196,547 (16.94)	206,385 (17.58)	211,683 (17.93)	218,054 (18.32)	222,333 (18.57)	227,398 (18.86)

**Table 2:** Relative change in DDI prevalence between 2013 and 2019 and the corresponding 95% CI.

DDI	2013	2019	Percent Change (95% CI)
	%	%	
Antidiabetics—beta-blocking agents	21.85	24.24	10.94 (9.57; 12.33)
Antidiabetics—fluoroquinolones	10.91	5.97	−45.25 (−46.5; −43.97)
ACEIs/ARBs—NSAIDs	27.87	21.54	−22.7 (−23.62; −21.77)
ACEIs/ARBs—potassium-sparing agents	8.28	7.45	−10.06 (−12.12; −7.95)
ACEIs/ARBs + diuretics—NSAIDs	13.38	9.37	−30.00 (−31.33; −28.65)
Diuretics—NSAIDs	24.66	17.71	−28.19 (−29.14; −27.24)
SSRIs—NSAIDs/ASA	13.61	11.76	−13.61 (−15.13; −12.05)
vitamin K antagonists—PPIs	10.75	5.64	−47.52 (−48.75; −46.26)
vitamin K antagonists—statins	8.38	4.79	−42.81 (−44.3; −41.29)
Clopidogrel—PPIs	7.09	8.11	14.36 (11.67; 17.12)
Corticosteroids—NSAIDs/ASA	14.24	13.36	−6.14 (−7.72; −4.53)

**Table 3:** Prevalence of DDIs involving antihypertensives and/or NSAIDs by year stratified by age classes. 2013–2019, Piedmont region (Italy).

		2013	2014	2015	2016	2017	2018	2019
	Age Group	%	%	%	%	%	%	%
**ACEIs/ARBs +** **potassium-sparing agents**	65–69 years	6.67	6.26	6.21	6.12	6.12	6.35	6.46
	70–74 years	7.31	7.23	6.79	6.64	6.56	6.48	6.80
	75–79 years	7.99	7.66	7.58	7.24	6.95	6.75	7.35
	80–84 years	9.37	9.04	8.72	8.07	7.85	7.59	7.93
	≥85 years	9.90	9.55	9.24	8.78	8.54	7.89	8.23
**ACEIs/ARBs +** **Diuretics—NSAIDs**	65–69 years	14.77	13.86	12.56	12.00	11.11	10.17	9.78
	70–74 years	14.81	14.13	13.19	12.62	11.72	10.59	10.42
	75–79 years	14.87	13.34	12.91	12.01	11.41	10.22	10.30
	80–84 years	12.63	12.07	11.09	10.77	10.28	9.56	9.36
	≥85 years	9.25	8.72	8.02	8.08	7.24	7.03	7.07
**ACEIs/ARBs +** **NSAIDs**	65–69 years	30.80	29.32	27.52	26.21	25.23	23.56	24.14
	70–74 years	30.17	28.54	27.37	26.81	25.63	24.00	24.37
	75–79 years	29.59	27.58	26.55	25.21	24.51	22.27	22.91
	80–84 years	26.74	25.26	23.98	23.05	22.34	20.34	21.03
	≥85 years	21.5	20.18	18.59	18.26	17.12	15.94	16.29
**diuretics + NSAIDs**	65–69 years	24.08	22.66	21.05	20.05	18.62	17.12	16.64
	70–74 years	24.73	23.39	22.10	21.18	19.93	18.29	18.18
	75–79 years	25.65	23.50	22.57	21.19	20.25	18.15	18.57
	80–84 years	25.00	23.75	22.22	21.02	19.98	18.03	18.22
	≥85 years	23.23	21.85	20.08	19.42	18.35	16.59	16.37
**corticosteroids + NSAIDs/ASA**	65–69 years	13.79	14.24	14.05	13.84	14.32	13.97	14.29
	70–74 years	14.19	14.16	13.97	14.06	14.07	13.72	14.24
	75–79 years	14.40	13.71	14.27	13.84	13.95	13.23	13.73
	80–84 years	13.97	13.33	13.56	13.08	13.16	12.69	13.11
	≥85 years	14.78	13.84	13.57	12.62	12.72	12.17	11.84
**SSRIs + NSAIDs/ASA**	65–69 years	11.54	11.22	10.73	10.66	10.42	10.08	10.15
	70–74 years	11.98	11.80	11.64	11.42	11.19	10.75	10.66
	75–79 years	13.47	13.06	12.77	12.70	12.40	12.03	11.56
	80–84 years	14.82	14.70	13.72	13.20	13.08	12.69	12.81
	≥85 years	16.10	15.39	14.71	13.87	13.97	12.91	12.85

## Data Availability

The pooled data that support the findings of this study are available from the corresponding author, L.S., upon reasonable request.

## Supplementary Materials

The following supporting information can be downloaded at: https://www.mdpi.com/article/10.3390/ijerph19127353/s1, Figure S1: Trend in the prevalence of other DDIs, overall and by sex. 2013–2019, Piedmont region (Italy); Table S1: List of ATC codes used to identify the DDI; Table S2: Prevalence of DDI by year stratified by age classes. 2013–2019, Piedmont region.
