# Supplementary material for: Time-Trends of Drug-Drug Interactions among Elderly Outpatients in the Piedmont Region (Italy): A Population-Based Study
Source: Int J Environ Res Public Health. 2022 Jun 15;19(12):7353. doi: 10.3390/ijerph19127353 (PMC9224286; doi:10.3390/ijerph19127353)
Supplement: Supplementary file 1 [file ijerph-19-07353-s001.zip › ijerph-1768327-supplementary.pdf]

Supplementary material

Table S1 – List of ATC codes used to identify the DDIs

Treatment	ATC code
antidiabetics	A10*
beta-blocking agents alone or in combination with other antihypertensive drugs	C07*
fluoroquinolones	J01MA*
ACEIs/ARBs alone or in combination with other antihypertensive drugs	C09*
NSAIDs	M01A*
potassium-sparing agents	C03D*
ACEIs/ARBs + diuretics	C03*, C07B*, C07C*,C07D*,C09BA*, C09DA*
diuretics alone or in combination with other antihypertensive drugs	C03*, C07B*, C07C*,C07D*,C09A*,C09B*, C09DA*
SSRIs	N06AB*
NSAIDs/ASA	M01A*, B01AC06
vitamin K antagonists	B01AA*
PPIs	A02BC*
statins alone or in combination with other lipid lowering drugs	C10AA*, C10BA*
clopidogrel	B01AC04
corticosteroids for systemic use	H02*

Figure S1 – Trend in the prevalence of other DDIs, overall and by sex. 2013-2019, Piedmont region

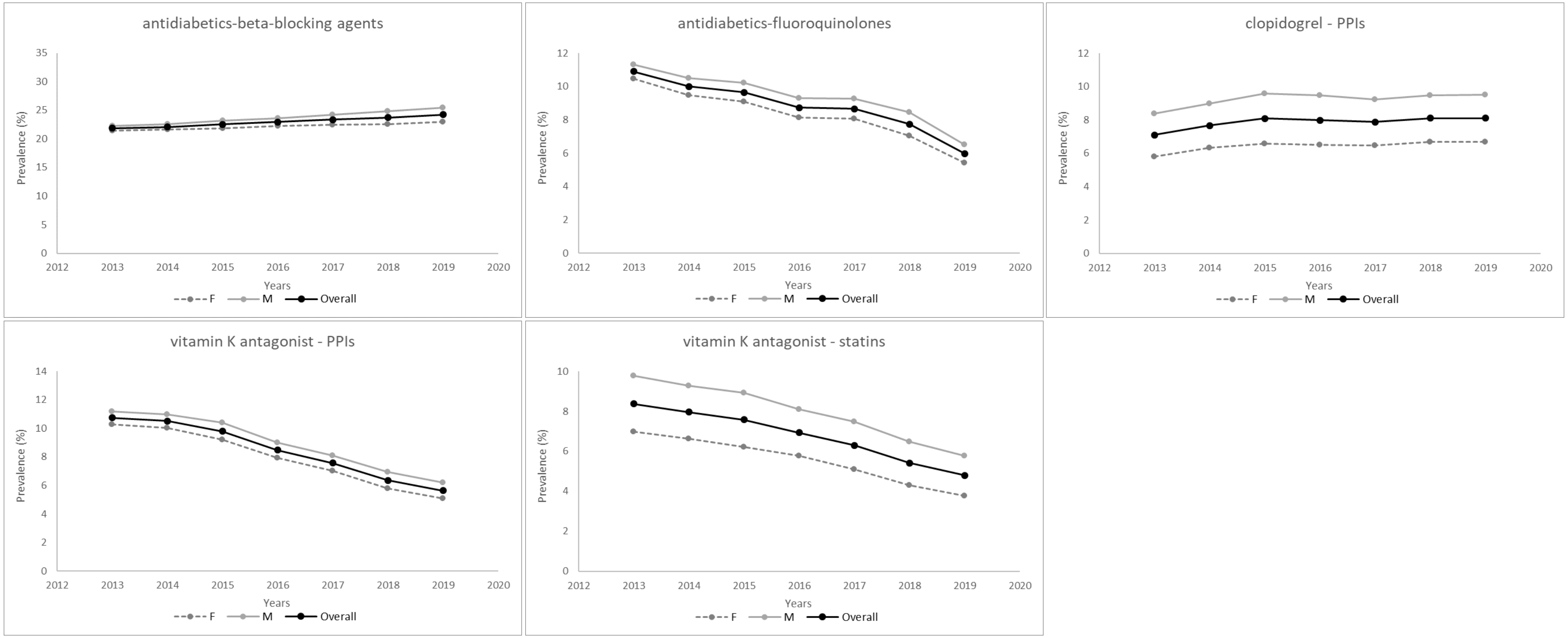

Table S2 Prevalence of other DDIs by year stratified by age classes. 2013-2019, Piedmont region.

		2013	2014	2015	2016	2017	2018	2019
	**Age group**	**%**	**%**	**%**	**%**	**%**	**%**	**%**
**antidiabetics+ beta-blocking agents**	65-69 years	27.94	27.79	28.27	28.43	28.57	28.99	29.12
	70-74 years	25.41	25.87	26.30	26.74	27.01	27.28	27.91
	75-79 years	22.98	23.47	23.59	24.25	24.54	24.81	25.55
	80-84 years	18.97	19.60	20.58	20.84	21.56	22.27	22.76
	≥85 years	14.18	14.35	15.01	15.86	16.72	17.37	17.91
**antidiabetics + fluoroquinolones**	65-69 years	12.20	11.68	11.12	9.85	9.87	8.72	6.63
	70-74 years	12.16	11.04	10.50	9.77	9.72	8.91	7.05
	75-79 years	11.19	10.37	10.16	9.16	8.89	8.27	6.33
	80-84 years	10.16	9.37	9.11	8.26	8.18	7.19	5.62
	≥85 years	8.78	7.72	7.61	6.91	7.08	6.04	4.53
**clopidogrel + PPIs**	65-69 years	6.97	6.87	7.22	7.18	6.64	7.19	7.11
	70-74 years	6.90	7.43	7.64	7.45	7.14	7.56	7.65
	75-79 years	7.01	7.66	8.08	8.01	8.01	8.01	8.09
	80-84 years	7.42	8.10	8.56	8.29	8.16	8.60	8.43
	≥85 years	7.13	8.10	8.73	8.76	8.94	8.75	8.85
**vitamin K antagonists + PPIs**	65-69 years	7.03	7.08	6.68	5.35	4.87	4.21	3.74
	70-74 years	9.46	8.82	8.25	7.12	6.03	5.08	4.45
	75-79 years	11.57	11.10	10.19	8.74	7.60	6.24	5.64
	80-84 years	13.40	13.10	11.98	10.44	9.16	7.55	6.44
	≥85 years	11.10	11.38	10.85	9.63	9.14	7.79	7.06
**vitamin K antagonists + statins**	65-69 years	6.72	6.11	6.06	5.04	4.68	3.93	3.49
	70-74 years	8.56	7.92	7.43	6.69	5.81	4.88	4.16
	75-79 years	9.81	9.19	8.69	7.88	6.96	5.98	5.34
	80-84 years	9.71	9.49	8.76	8.20	7.64	6.32	5.52
	≥85 years	5.92	6.07	6.16	5.95	5.64	5.19	4.78
